# Impact of ovotransferrin on the membrane integrity of *Salmonella* Enteritidis under egg-white conditions

**DOI:** 10.3389/fmicb.2025.1539663

**Published:** 2025-01-17

**Authors:** Julie Legros, Sylvie Bonnassie, Marie-Françoise Cochet, Sophie Jan, Simon C. Andrews, Florence Baron

**Affiliations:** ^1^STLO, INRAE, Institut Agro, Rennes, France; ^2^School of Biological Sciences, Health & Life Sciences Building, University of Reading, Reading, United Kingdom; ^3^UFR Sciences de la vie et de l’environnement, Université de Rennes I, Rennes, France

**Keywords:** *Salmonella* Enteritidis, ovotransferrin, egg white, membrane permeabilization, membrane potential

## Abstract

**Introduction:**

Eggs can mediate foodborne disease resulting in salmonellosis outbreaks that are most commonly caused by *Salmonella enterica* serovar Enteritidis. Ovotransferrin is a prominent egg-white antimicrobial glycoprotein belonging to the transferrin family, members of which exhibit powerful iron-chelating activity. However, several studies have also described the ability of transferrin proteins to disrupt bacterial membranes. This study aimed to investigate the antimicrobial activity of ovotransferrin toward *S.* Enteritidis membranes at 30°C under egg-white conditions.

**Materials and methods:**

This aim was supported by the deployment of a synthetic medium designed to mimic egg-white (matching the ionic composition and pH). The ability of ovotransferrin to induce bacterial membrane permeabilization in *S.* Enteritidis was investigated by measuring substrate accessibility to periplasmic *β*-lactamase and cytosolic *β*-galactosidase.

**Results:**

The depolarization of the inner membrane of *S.* Enteritidis was measured using a fluorescence probe [DiSC_3_(5)]. The results show that ovotransferrin induces permeabilization of the outer membrane but not the inner membrane whereas egg white permeabilizes both membranes. In addition, the dissipation of the proton motive force by egg white was found to involve a contribution by ovotransferrin since this protein provoked inner-membrane depolarization.

**Discussion:**

It can thus be concluded that ovotransferrin exerts a membranes perturbation activity on S. Enteritidis under egg-white conditions, in addition to its well-known iron-chelation activity.

## Introduction

Consumption of raw or undercooked egg or egg products increases the risk of foodborne disease. In 2023, *Salmonella* in “eggs and egg products” caused the largest number of outbreaks among all agent/food pair, mostly involving the pathogen *Salmonella enterica* serovar Enteritidis (European Food Safety Authority and European Centre for Disease Prevention and Control, 2024). Egg contamination can occur upon contact with microorganisms present in the laying environment, after handling or during the storage of eggs (horizontal transmission). Contamination can also take place during egg development in the uterus or oviduct of an infected hen (vertical transmission) (Gantois et al., 2009). To counter such bacterial contamination issues, eggs utilize numerous defenses, such as the eggshell barrier and the antimicrobial characteristics of the egg white (EW). Due to varying levels of ovomucin, EW is composed of thick and thin layers that exhibit higher and lower degrees of viscosity, respectively; this viscosity reduces the motility of bacteria restricting their accessibility to nutrients. In addition, the alkaline pH of EW (which shifts to pH 9.3 a few days after laying) contributes to bacterial growth restriction. Furthermore, EW contains a high protein content (10.7% w/v) and some of the proteins within EW have antimicrobial properties (Réhault-Godbert et al., 2019).

Ovotransferrin (OT) is one such antimicrobial EW protein. It is the most abundant antimicrobial protein of EW (13 g/L) and, importantly, it exhibits antibacterial activity (Réhault-Godbert et al., 2019). OT belongs to the transferrin family, members of which function as high-affinity ferric-iron-binding proteins. OT consists of two homologous lobes (the C-and N-terminal lobes) and each is divided into two subdomains (C1 and C2, and N1 and N2, respectively) (Kurokawa et al., 1999). Each lobe carries a single ferric-iron-binding site, with affinity constants of 1.5 × 10^14^ and 1.5 × 10^18^ M^−1^ for the N-and C-lobes, respectively. Due to its iron-chelating activity, OT generates an iron-deficient environment for bacterial growth in EW and several studies have shown that the addition of iron enhances bacterial growth in EW (Baron et al., 1997; Garibaldi, 1970; Lock and Board, 1992). Thus, the iron restriction imposed by OT appears responsible for the antimicrobial activity of EW. However, several studies have also reported that OT causes damage to microbial membranes (Aguilera et al., 2003; Ellison et al., 1988). Indeed, an early report of the antimicrobial activity of OT indicates that a direct interaction is required for an antimicrobial effect on *Candida albicans* (Valenti et al., 1985). Subsequently, it was shown that the OT homologues, lactoferrin and transferrin, induce lipopolysaccharide (LPS) release in *Escherichia coli* and *Salmonella Typhimurium*. Iron saturation of the proteins inhibited this activity (Ellison et al., 1988) indicating that the metal chelating activity of lactoferrin and transferrin is responsible for this effect. Indeed, LPS is stabilized by its association with divalent cations, and it was subsequently shown that the LPS-release activity of lactoferrin and transferrin is mediated by sequestration of Mg^2+^ and Ca^2+^ (Ellison et al., 1990). In *E. coli*, OT is able to both permeabilize the outer membrane and access the inner membrane where it induces leakage of K^+^ ions (Aguilera et al., 2003). This selective ion leakage results in dissipation of the electrical potential of the inner membrane as well as a decrease of the proton motive force (Aguilera et al., 2003).

In the context of EW, Baron et al. (2014) showed that OT induces depolarization of the inner membrane of *Bacillus cereus* and suggested that this membrane perturbation contributes to the lysis of *B. cereus* that is observed upon exposure to EW at 30°C. Furthermore, a transcriptomic study using microarrays and qRT-PCR showed that EW proteins of >10 kDa (which include OT) up-regulate the *psp* genes in *Salmonella* Enteritidis (induced by membrane damage and loss of the proton motive force; Joly et al., 2010) and also provoke the inner-membrane depolarization (Baron et al., 2020). These findings are consistent with a potential role for OT in inner-membrane damage. Therefore, the aim of this study is to further understand the ability of OT to disrupt the membranes of *S.* Enteritidis under EW conditions. The temperature of egg storage varies, from refrigerated to ambient temperatures, depending on climate and/or the regulations applied by different countries. The activity of egg-white against *S.* Enteritidis is temperature dependent: at refrigeration temperatures, no growth is observed; between 20 and 30°C, *S.* Enteritidis can exhibit weak growth; at 37°C, egg white has a bacteriostatic effect; whereas at 42°C, a bactericidal effect is observed (see Baron et al., 2016 for review). Here, we investigated the membrane perturbating activity of OT, and its contribution to the antibacterial power of egg white, at a temperature (30°C) that allows *Salmonella* to exhibit growth, albeit slight.

## Materials and methods

### Bacterial strain

The strain of *Salmonella enterica* subsp*. enterica* serovar Enteritidis (*S.* Enteritidis) used in this study was NCTC13349, kindly provided by Matthew McCusker (Centre for Food Safety and Foodborne Zoonomics, Veterinary Sciences Centre, University College, Dublin, Ireland). This strain was transformed with plasmid pBBC129 for permeability experiments in order to generate *S.* Enteritidis pBBC129 which produces periplasmic *β*-lactamase and inner *β*-galactosidase (as *S.* Enteritidis lacks the corresponding genes).

Plasmid pBBC129 was generated as follows. The *lacZ* gene of *E. coli* MG1655 (F^−^, *λ^−^, ilvG*^−^, *rfb*-50, *rph*-1) (Blattner et al., 1997) was amplified by PCR using the Fidelio polymerase (Ozyme, Saint-Cyr-L’Ecole, France), and the primers TAGCTCACTCATTAGGCAC and AATGGATTTCCTTACGCG, according to the manufacturer’s instructions. The amplified *lacZ* PCR fragment (3,226 bp) was purified using a QIAquick gel extraction kit (Qiagen, Les Ulis, France) and ligated with *Eco*RV-digested pBR322 plasmid DNA (Bolivar et al., 1977). The ligation mixture was used to transform *E. coli* DH5α (F^−^, *endA1, glnV44, thi-1, recA1, relA1, gyrA96, deoR, nupG, purB20,* φ80d*lacZΔM15* Δ (*lacZYA-argF*)*U169, hsdR17*(rK^−^ mK^+^), *λ^−^*) (Meselson and Yuan, 1968) by electroporation. Lac^+^ Amp^R^ transformants were selected on LB agar containing ampicillin (100 μg/mL), 40 μM X-gal and 1 mM isopropyl-*β*-D-thiogalactopyranoside (IPTG; Sigma, Saint Quentin-Fallavier, France). Plasmid DNA was then extracted (QIAprep Spin Miniprep Kit, Qiagen), and the identity of the isolated plasmid (designated pBBC129) was verified by restriction digestion analysis. *S.* Enteritidis NCTC13349 was transformed with pBBC129 to generate *S.* Enteritidis pBBC129. This strain was used to study outer-and inner-membrane permeabilization. The behavior of this strain in the various media used in this study was determined and was found to be comparable to that of *S.* Enteritidis NCTC13349 (data not shown).

Before each experiment, *S.* Enteritidis strains were propagated overnight at 37°C in Tryptone Soy Broth (TSB, Biokar diagnostics, Beauvais, France), and then re-propagated for a further overnight period in the same conditions. For *S.* Enteritidis pBBC129, 100 μg/mL ampicillin (Sigma) was included in the TSB medium.

### Preparation of sterile egg white

EW was prepared from eggs obtained from a conventional hen housing system according to Baron et al. (1997). Eggshell surfaces were sanitized with absolute alcohol. Residual alcohol was removed by briefly flaming the eggshell. Eggshells were then broken, under sterile conditions, and the collected EW was aseptically homogenized with an Ultra-Turrax^®^ Disperser DI25 Basic (Ika, Grosseron, Saint-Herblain, France) for 1 min at 9500 × rpm. The pH of the isolated EW was 9.3 ± 0.1 and its sterility was confirmed by the absence of growth (for 24 h at 37°C) following addition of 1 mL EW into 20 mL of melted Tryptone Soya Agar (TSA; Biokar Diagnostics, Pantin, France) in a Petri dish.

### Preparation of egg-white filtrate

EWF was prepared by ultrafiltration using a pilot unit (TIA, Bollène, France) equipped with an Osmonics membrane (5.57 m^2^, 10 kDa cut-off; PW 2520F, Lenntech BV, Delft, Netherlands), according to Baron et al. (1997). EWF was sterilized by filtration (Nalgene^®^ filter unit, pore size <0.2 μm, Osi, Elancourt, France) and then stored at 4°C until use. EWF has the same pH and ionic strength as EW but lacks EW macromolecules above 10 kDa. The composition of EWF was determined by Cochet et al. (2021) and is provided in Table 1.

**Table 1:** Comparison of the ionic compositions of EW, EWF, and SM.

	Minimal concentration in EW (mM)*	Maximal concentration in EW (mM)*	Concentration in 10 kDa EWF (mM)**	Concentration in SM (mM)
Sodium	67.424	80.908	96.1	59
Sulfur	50.834	56.136	0.69	1
Chloride	–	49	–	44.5
Potassium	35.807	44.247	44.7	40
Total *N*	–	1,364	2.3	2
Phosphorus	4.1971	7.1028	1.7	2
Magnesium	3.7029	4.9372	3.32	2
Calcium	1.2476	2.9942	0.96	0.25
Iron	0.0036	0.0179	<0.00002	–
Zinc	0.0015	0.0185	0.0005	–
Copper	0.0029	0.0058	0.00098	–
Manganese	0.0013	0.0020	0.00002	–
Glucose	–	25	21.5	20

* Values were obtained from Ciqual (2024), Nau et al. (2010), Nys and Sauveur (2004), Sauveur (1988), and Stadelman and Cotterill (1995). ** Values were obtained from Cochet et al. (2021).

### Design and preparation of synthetic medium

SM was developed according to the ionic composition of EW and EWF (Table 1) whilst accounting for differences with EWF resulting from chemical elements associated with proteins that are removed during ultrafiltration with a 10 kDa cut-off membrane. As the pH of EW reaches ~9.3 a few days after laying this was an important parameter considered in the design of the SM. Thus, through the inclusion of a 55 mM sodium carbonate/bicarbonate buffer, a pH of 9.3 was obtained. Moreover, this buffer provides a source of sodium and carbonate/bicarbonate ions. This concentration corresponds to the concentration of bicarbonate present in EW, as derived from the hen’s blood and transferred during hydration of EW in the hen’s uterus. In addition, the bicarbonate is also required for the binding of iron to OT (Phelps and Antonini, 1975). Sulfur is the second most abundant element found in EW (50.8 to 56.1 mM), but almost all the sulfur in EW is protein associated, which explains its 78-fold lower concentration in EWF (0.69 mM). Thus, in SM, sulfur was supplied with a concentration of 1 mM in the form of (NH_4_)_2_SO_4_. Chloride is present in EW at a concentration of 49 mM. Thus, KCl, MgCl_2_ and CaCl_2_ were the three forms of chloride included in the SM for achieving the final concentration of 44 mM. These three chemicals also provide a source of potassium (40 mM), magnesium (2 mM) and calcium (0.25 mM) in SM. In EW, the total nitrogen concentration is very high (1.36 M), but as for sulfur, most of the EW nitrogen is provided in the form of protein. Thus, the ultrafiltration of EW resulted in an ~600-fold reduction in nitrogen concentration in the resulting EWF (to 2.3 mM). For this reason, the nitrogen source in SM was provided by ammonium in the form of 2 mM (NH_4_)_2_SO_4_. The concentration of phosphorus is between 4.2 and 7.1 mM in EW and is partially protein-bound. Thus, a concentration of 2 mM phosphorus in the form of NaH_2_PO_4_ was included in SM, a concentration that matches that of the EWF. In EW and EWF, the concentration of magnesium is almost the same (between 3 and 4 mM); a source of Mg was provided in SM by the inclusion of 2 mM MgCl_2_. The concentration of calcium in EW is between 1.3 and 3 mM, and is 0.96 mM in EWF. Only a 0.25 mM final concentration of calcium was included in SM since precipitation occurred at higher concentrations. This concentration is slightly higher than that of the M9 minimal medium (i.e., 0.1 mM). Iron, zinc, copper and manganese are present in trace amounts in EW (between 1.5 and 1.85 μM) and in EWF (between <0.02 and 0.98 μM), so these elements were not specifically added to SM as they were already present in trace amounts in SM. A source of sugar is essential for bacterial growth. In EWF, like in EW, glucose is the dominant form of carbohydrate with a concentration of ~20 mM. The same quantity was therefore provided in the SM.

SM (pH was 9.3 ± 0.1) was thus prepared by adding the following, in the order given, which were dissolved in ultrapure water: 1 mM (NH4)_2_SO_4_ (Panreac, Barcelona, Spain), 2 mM Na_2_HPO_4_ (Merk, Darmstadt, Germany), 40 mM KCl (Merck), 20 mM glucose (Merck), 2 mM MgCl_2_ (Merck), 0.25 mM CaCl_2_ (Sigma) and 55 mM sodium carbonate buffer (pH 9.3). Finally, the SM was sterilized by filtration (Nalgene^®^ filter unit, pore size <0.2 μm) (see Table 1).

### Preparation of ovotransferrin solutions

Apo-OT was purchased from Sigma and is substantially iron free. OT was prepared in EWF or in SM at concentrations ranging from 1.3 to 13 g/L, corresponding to 10 and 100% of the theoretical concentration found in EW. For some experiments, ferric citrate (Sigma) was added to obtain a theoretical OT saturation from 10 (33.4 μM) to 110% (367 μM). The OT solutions were sterilized by filtration, as above.

### Growth studies

*Salmonella* Enteritidis growth was compared after 24 h incubation at 30°C in: EW, EWF, SM or EWF; with or without 10% EW, 1.3 g/L of apo-OT, 13 g/L of OT iron saturated (from 0 to 110% of theoretical saturation) OT. *S.* Enteritidis NCTC13349 was twice passaged overnight at 37°C in TSB and then centrifuged for 7 min at 5,600 g and 15°C. The cell pellets were washed three times in tryptone salt medium (TS, 8.5 g/L NaCl, 1 g/L Tryptone). Bacterial cells were then diluted and inoculated at 2% of the original volume in a final volume of 800 μL of the corresponding medium, to give 5 log_10_ CFU/mL. The diluted cultures were then propagated in a 96-well plates of 2.2 mL well volume (Starlab, Bagneux, France) with 800 μL applied per well. Enumeration of *S.* Enteritidis cells was performed after 24 h of incubation at 30°C in TSA, using a rapid and cost-effective method previously described by Baron et al. (2006). Experiments were performed with three biological replicates and three technical replicates. Statistical analysis was performed using one-way ANOVA followed by a Tukey Contrast test, using R software (version 1.4.1106).

### Membrane permeabilization

Membrane permeabilization was determined spectroscopically using a method adapted from Lehrer et al. (1988) and Derde et al. (2013). As previously described for *E. coli* ML-35p, *β*-lactamase and *β*-galactosidase activities were measured to detect outer-and inner-membrane permeabilization, respectively (Derde et al., 2013; Lehrer et al., 1988). For this experiment, *S.* Enteritidis pBBC129 was used to allow production of *β*-lactamase and *β*-galactosidase.

After propagation as indicated above, the culture was centrifugated at 7,000 x g for 7 min at 15°C, washed three times with TS and then the OD_600nm_ (Colourwave Colorimeter WPA CO7500, Biochrom, Holliston, United States) was adjusted to 0.35 in the same medium. TS medium (270 μL) containing the *β*-lactamase substrate, nitrocefin (Sigma), at 30 mM or the *β*-galactosidase substrate, ortho-nitrophenyl-*β*-galactopyranoside (ONPG), at 2.5 mM (Sigma), prepared in 10 mM phosphate buffer (pH 7.4), was applied to a 96-well microtiter plate. Then, 30 μL of bacterial suspension were added. Polymyxin B (50 μg/mL) (Sigma), a polypeptide antibiotic, was used as a positive control for the permeabilization of the outer membrane. Melittin (15 μg/mL) (Sigma), an amphipathic cytotoxin derived from bee venom, was used as a positive control for the permeabilization of the inner membrane. The plate was then incubated in a Spectramax M2 spectrophotometer (Molecular Devices, San Jose, United States) at 30°C for 5 h. Absorbance values were read at 490 and 405 nm to measure HP nitrocefin release (for outer-membrane permeabilization) and ONP production (for inner-membrane permeabilization), respectively. According to Derde et al. (2013), the intersection of the baseline and the tangent to the curve at the point where the rate is maximal (defined as “Vmax” here) were used to calculate the lag time (delay between outer-and inner-membrane permeabilization). The enzymatic reaction rate was determined using the following formula, ∆A_nm_/30 min*10^3^, and was expressed in milliA_nm_/min. Experiments were performed with three biological and three technical replicates. Statistical significance was determined as indicated above.

### Membrane depolarization

A spectrofluorescent method adapted from Epand et al. (2010) was used to detect depolarization of the inner membrane of *S.* Enteritidis. A change in membrane potential was detected using 3,3′-diopropylthiadicarbocyanine iodide (DiSC_3_(5), Sigma), a lipophilic potentiometric dye. Bacteria were grown as previously described, and then ten-fold diluted in TSB and incubated for 3.5 h at 37°C. Bacteria were centrifuged (5,600 g for 7 min at 15°C) and washed three times in HEPES buffer (5 mM HEPES, 5 mM glucose, pH 7.2) before resuspension in the same buffer to give an OD_600nm_ of 0.5. The cells were then charged using a 20 μM DiSC_3_(5) solution and 100 mM KCl, to balance cytoplasmic and external K^+^ concentrations. The mix was incubated for 15 min at 37°C in the dark to stabilize the dye signal. Then, cells charged with DiSC_3_(5) were diluted 10 times in various media and incubated for 10 min at 30°C. Fluorescent measurements corresponding to the disruption of the membrane potential gradient (ΔΨ) were made using a spectrofluorometer (Molecular Devices Spectra, MAX Gemini XS) at an excitation and emission wavelengths of 622 and 670 nm, respectively. Three biological and technical replicates were performed. Results are expressed in relative fluorescent units (RFU). Statistical analysis was determined as above.

### Scanning electron microscopy

The cell surface morphologies of *S.* Enteritidis were compared after 24 h incubation at 30°C in TSB, SM or SM with apo-OT (13 g/L). *S.* Enteritidis NCTC13349 was twice passaged overnight at 37°C in TSB and then centrifuged for 7 min at 5600 g and 15°C. The cell pellets were washed three times in TS. Bacterial cells were then diluted and inoculated at 2% in SM or in SM with apo-OT (13 g/L) to give an initial inoculum of 7 log_10_ CFU/mL. After 24 h incubation at 30°C, 2 mL of each cell suspension were placed onto a 0.20 μm pore-size membrane filter (Millipore-Isipore, Merck) and fixed for 1 day in 0.1 M sodium cacodylate buffer (pH) containing 2.5% glutaraldehyde (Sigma-Aldrich). Samples were then washed four times for 15 min in 0.1 M sodium cacodylate buffer and postfixed for 1 h with 0.1 M sodium cacodylate buffer containing 1% OsO_4_ (Euromedex). The samples were then dehydrated with a graded ethanol series (10, 25, 50, 75 and 95% ethanol; 15 min at each concentration) followed by treatment with 100% ethanol for 2 h. Samples were then metallized with gold palladium (Sigma-Aldrich). Microscopy was performed with a Jeol-6400F microscope (Olympus) and electron microscopy images were taken at 7 kV. The lengths of 30 cells for each condition (TSB, SM and SM with apo-OT at 13 g/L) were measured and statistical analysis was performed as above.

## Results

### Assessing the influence of egg white on *Salmonella* Enteritidis using synthetic medium

Studying the antimicrobial impact of a particular EW protein (such as OT) under EW conditions is a challenge largely due to the complex physical and chemical properties of EW, and because of the presence of numerous proteins. One solution to this problem has been the use of EWF, generated by ultrafiltration of EW using a 10 kDa cut-off membrane (Baron et al., 1997) thus removing macromolecules >10 kDa but preserving the pH and ionic composition of EW. However, EWF is not peptide-free and a recent study has highlighted the presence of antimicrobial <10 kDa peptides in this medium (Cochet et al., 2021) which could potentially interfere with OT activity.

Since no suitable EW synthetic medium was available, a medium (designated SM; see Methods and Table 1), closely-matching the ionic composition and pH of EW, was developed using the ionic compositions of EW and EWF as guides (Ciqual, 2024; Cochet et al., 2021; Nau et al., 2010; Nys and Sauveur, 2004; Sauveur, 1988; Stadelman and Cotterill, 1995).

**Figure 1:**
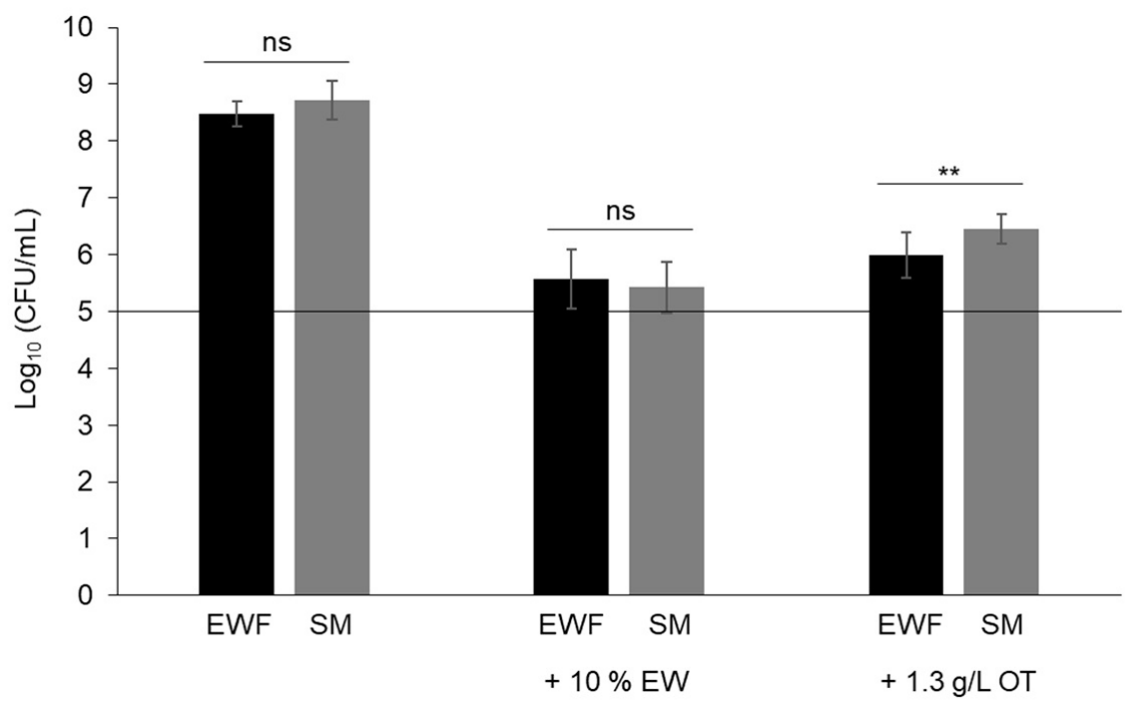
Comparison of the growth of *S.* Enteritidis in SM and EWF, with and without EW and apo-OT. *S.* Enteritidis (at a starting cell density of 5 log_10_ CFU/mL represented by the horizontal line) was incubated at 30°C for 24 h in SM or EWF, with or without 10% EW or apo-OT at 1.3 g/L. Error bars indicate standard deviations from the mean derived from one to three biological experiments, with three technical replicates Significant differences between each condition were assessed using one-way ANOVA followed by a Tukey contrast test. The “ns” designation indicates “no significant difference” with a *p*-value >0.05 and ** indicates a significant difference with a *p*-value <0.01.

In order to consider the suitability of SM as a model EW medium, *S.* Enteritidis growth was compared in SM and EWF over a 24 h period at 30°C (a temperature that allows growth in EW and EWF) in SM with/without 10% EW. The egg-based media, EWF and EWF with 10% EW, were used as controls. The results showed that *S.* Enteritidis growth is similar in EWF and SM (i.e., cell number increased by 3.5 and 3.6 log_10_ CFU/mL, respectively) (Figure 1). A growth reduction of ~1,000 fold for *S.* Enteritidis was observed in both media after addition of 10% EW (i.e., cell number increased by only 0.4 and 0.9 log_10_ CFU/mL for SM and EWF, respectively). These values are similar to those obtained after 24 h incubation in EW at 30°C (i.e., cell number increased by 0.3 log_10_ CFU/mL, data not shown). A growth reduction of ~100 fold for *S.* Enteritidis was observed in both media after addition of apo-OT (1.3 g/L; i.e., at 10% of its concentration in EW). *S.* Enteritidis growth after addition of apo-OT was only slightly, although significantly, higher in SM than to EWF (i.e., cell number increased by 1.6 and 1.1 log_10_ CFU/mL, respectively). The results thus indicate that the antimicrobial activity of EW and OT are exhibited to similar degrees in both SM and EWF. The antimicrobial effect of EW, and EWF with 10% EW, on *S.* Enteritidis have been previously reported by Baron et al. (1997, 2017, 2020). The slight difference in the impact of apo-OT (at 1.3 g/L) on growth in SM with respect to that seen in EWF may be caused by the presence of antimicrobial peptides in EWF (Cochet et al., 2021), which are not present in SM. In summary, these results indicate that SM is a suitable medium for studying the impact of OT on *S.* Enteritidis in conditions mimicking EW (i.e., same pH and ionic composition) and offers an advantage over EWF since it is entirely free of proteins or peptides.

### Impact of the iron chelation activity of ovotransferrin on *Salmonella* Enteritidis growth

**Figure 2:**
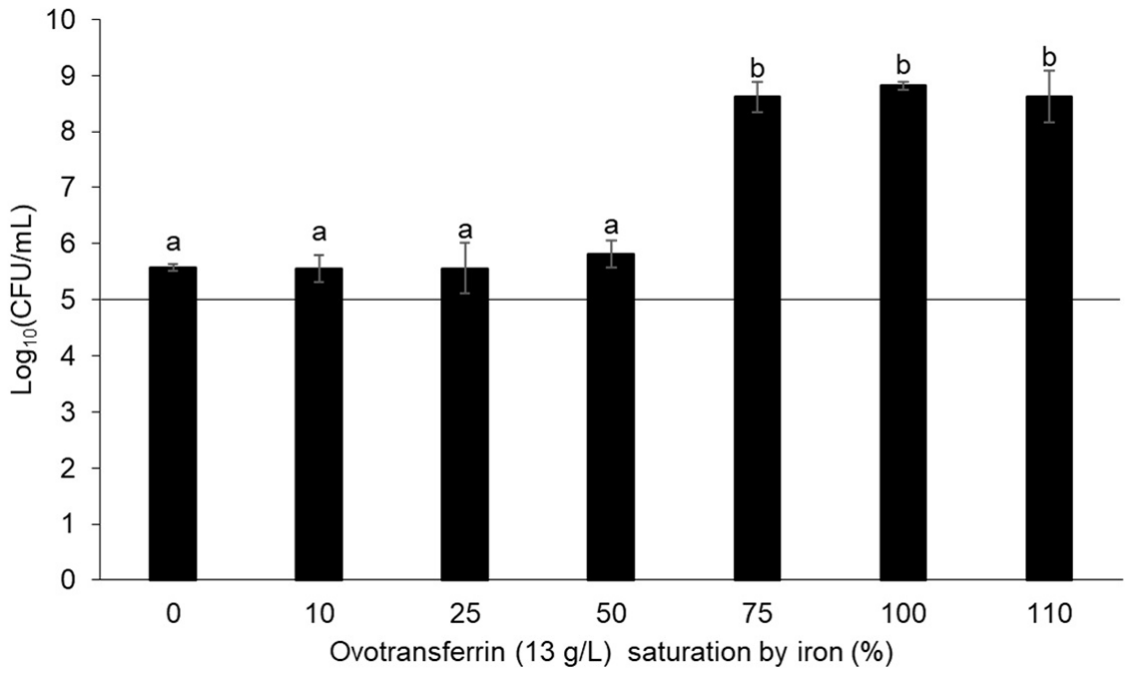
Impact of the iron-chelating activity of OT on *S.* Enteritidis growth in SM. *S.* Enteritidis NCTC13349 (at a starting cell density of 5 log_10_ CFU/mL represented by the horizontal line) was incubated at 30°C for 24 h in SM with 13 g/L OT at 0 to 110% iron saturation (using iron citrate). Error bars indicate standard deviations from the mean derived from one biological experiment, with three technical replicates. Significant differences between each condition were assessed using a one-way ANOVA test followed by a Tukey test with R software (version 1.4.1106). Several significantly different groups were identified by a letter, with a *p*-value <0.05.

The antibacterial activity of OT is considered to be mediated by the iron deficiency that it imposes upon EW. This view is supported by numerous studies demonstrating that iron supplementation promotes bacterial growth in EW (Baron et al., 1997; Garibaldi, 1970; Lock and Board, 1992). To determine whether OT might have an antibacterial action beyond iron restriction, the contribution of the iron-restriction activity of OT to the antibacterial activity of EW was further investigated in this study. Thus, *S.* Enteritidis was incubated in SM with 13 g/L of iron-saturated (0 to 110%) OT. After 24 h at 30°C, *S.* Enteritidis was subject to a substantial growth restriction by provision of OT iron-saturated at 0 to 50% (i.e., cell number increased by just 0.4–0.7 log_10_ CFU/mL) (Figure 2). However, at above 50% OT iron-saturation, *S.* Enteritidis growth was little impacted by OT (i.e., cell number increased by between 3.5–3.7 log_10_ CFU/mL) (Figure 2). These results confirm that the iron-chelating activity of OT is responsible for the restriction of *S.* Enteritidis growth at 30°C, under EW conditions. It should be noted that OT suppressed growth in SM more severely when supplied at 13 g/L than at 1.3 g/L (growth increased by 0.4 ± 0.06 and 1.6 ± 0.14 log_10_ CFU/mL, respectively; Figures 1, 2), indicating a clear concentration-dependent effect.

### Effect of ovotransferrin under egg-white condition on permeabilization of the *Salmonella* Enteritidis inner and outer membranes

The effect of OT’s iron-restriction impact on the growth of *S.* Enteritidis is well established in the literature, but this is not the case for any effect that OT may have on the membrane integrity of *S.* Enteritidis. Thus, to explore whether OT can induce permeabilization of the outer and/or inner membranes of *S.* Enteritidis, a strain (*S.* Enteritidis pBBC129) expressing *β*-lactamase and *β*-galactosidase was generated and deployed. This enabled measurement of substrate accessibility to *β*-lactamase and *β*-galactosidase in the periplasm and cytoplasm, respectively, in response to outer-and inner-membrane permeabilization, respectively (Derde et al., 2013; Lehrer et al., 1988).

**Figure 3:**
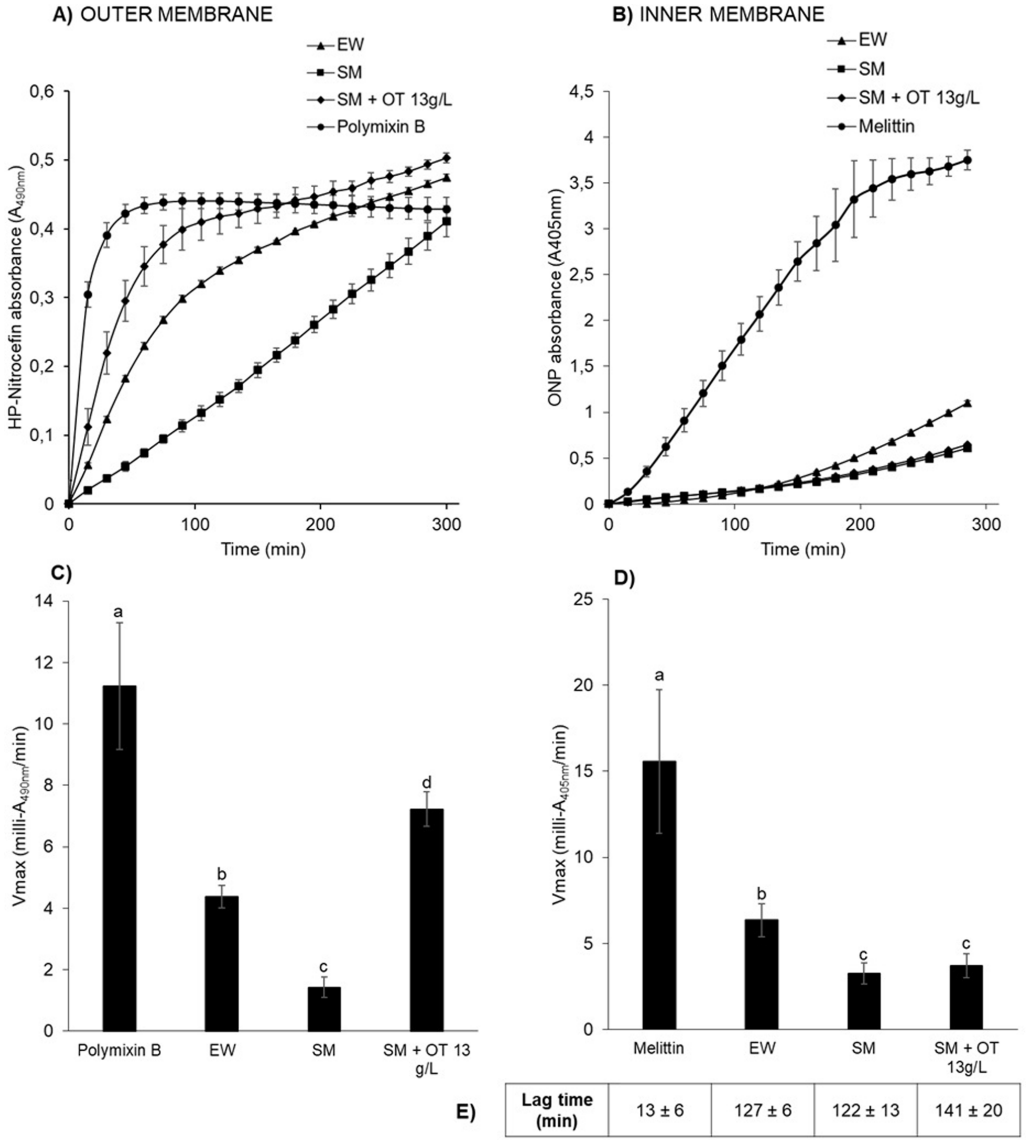
*S.* Enteritidis membrane permeabilization by EW and apo-OT. Monitoring of the permeabilization of the outer **(A)** and inner **(B)** membrane of *S.* Enteritidis (grown to an OD_600nm_ of 0.35) in EW (triangles), SM (squares), SM with 13 g/L of apo-OT (diamonds) and polymyxin B (50 μg/mL) or melittin (15 μg/mL) (circles) positive controls, respectively. Comparison of the Vmax for outer **(C)** and inner **(D)** membranes with polymyxin B or melittin, respectively; EW; SM and SM with 13 g/L of apo-OT (SM + OT 13 g/L). Additionally, the lag time, corresponding to the delay between the outer and inner membrane permeabilization, was calculated for each medium **(E)**. Each data point represents an average of three biological experiments with three technical replicates, with standard error indicated. Significant differences between each condition were assessed using a one-way ANOVA test followed by a Tukey test with R software (version 1.4.1106). The significantly different groups are identified by distinct letters, with a *p*-value <0.05.

The results show that outer-and inner-membrane permeabilization progressed steadily with incubation time as *S.* Enteritidis was exposed to the different conditions at 30°C (Figures 3A,B). For outer-membrane permeabilization (Figures 3A,C), the peptide-antibiotic polymyxin B was used as a positive control. Within the first few minutes of exposure to polymyxin B, *β*-lactamase activity increased rapidly (Vmax of 11.2 ± 2.1 milli-A_490nm_/min), indicating rapid permeabilization of the outer membrane of *S.* Enteritidis (Figures 3A,C). *β*-Lactamase activities were also observed upon EW exposure, but significant differences were observed with respect to the other media employed. In SM, the *β*-lactamase activity was significantly lower (Vmax of 1.4 ± 0.3 milli-A_490nm_/min) than in EW (Vmax of 4.4 ± 0.4 milli-A_490nm_/min) or in SM with 13 g/L of apo-OT (Vmax of 7.2 ± 0.6 milli-A_490nm_/min) (Figures 3A,C). These results indicate that the *S.* Enteritidis outer membrane is permeabilized by both EW and apo-OT exposure, and that apo-OT (at the concentration corresponding to that in EW) provokes a more drastic permeabilization than EW with a significant difference between EW and SM containing 13 g/L of apo-OT (Vmax of 4.4 ± 0.4 and 7.2 ± 0.6 milli-A_490nm_/min, respectively). This difference may well be explained by the presence of proteins or peptides in EW that could reduce the impact of OT on the *S.* Enteritidis membrane.

For the inner-membrane permeabilization studies, melittin (an amphipathic cytotoxin derived from bee venom), was used as a positive control. A high *β*-galactosidase activity was observed in the presence of melittin (Vmax of 15.6 ± 4.2 milli-A_405nm_/min) along with a relatively short lag time (13 ± 6 min corresponding to the delay between the outer and inner membrane permeabilization), indicating a major permeabilization of the inner membrane (Figures 3B,D). EW also induced a permeabilization of the inner membrane, but this was 2.5-fold weaker than seen for melittin (Vmax of 6.3 ± 0.9 milli-A_405nm_/min; lag time of 127 ± 6 min) (Figures 3B,D). Inner-membrane permeabilization was significantly higher in EW than in SM or in SM with apo-OT at 13 g/L (Vmax of 3.2 ± 0.6 and 3.7 ± 0.7; lag times 122 ± 13 min and 141 ± 20 min, respectively) (Figures 3B,D), and there was no significant difference between SM and SM with apo-OT at 13 g/L. These results indicate that OT does not lead to inner-membrane permeabilization and suggest that other EW-proteins are responsible for the weak inner-membrane disrupting effect observed in EW.

**Figure 4:**
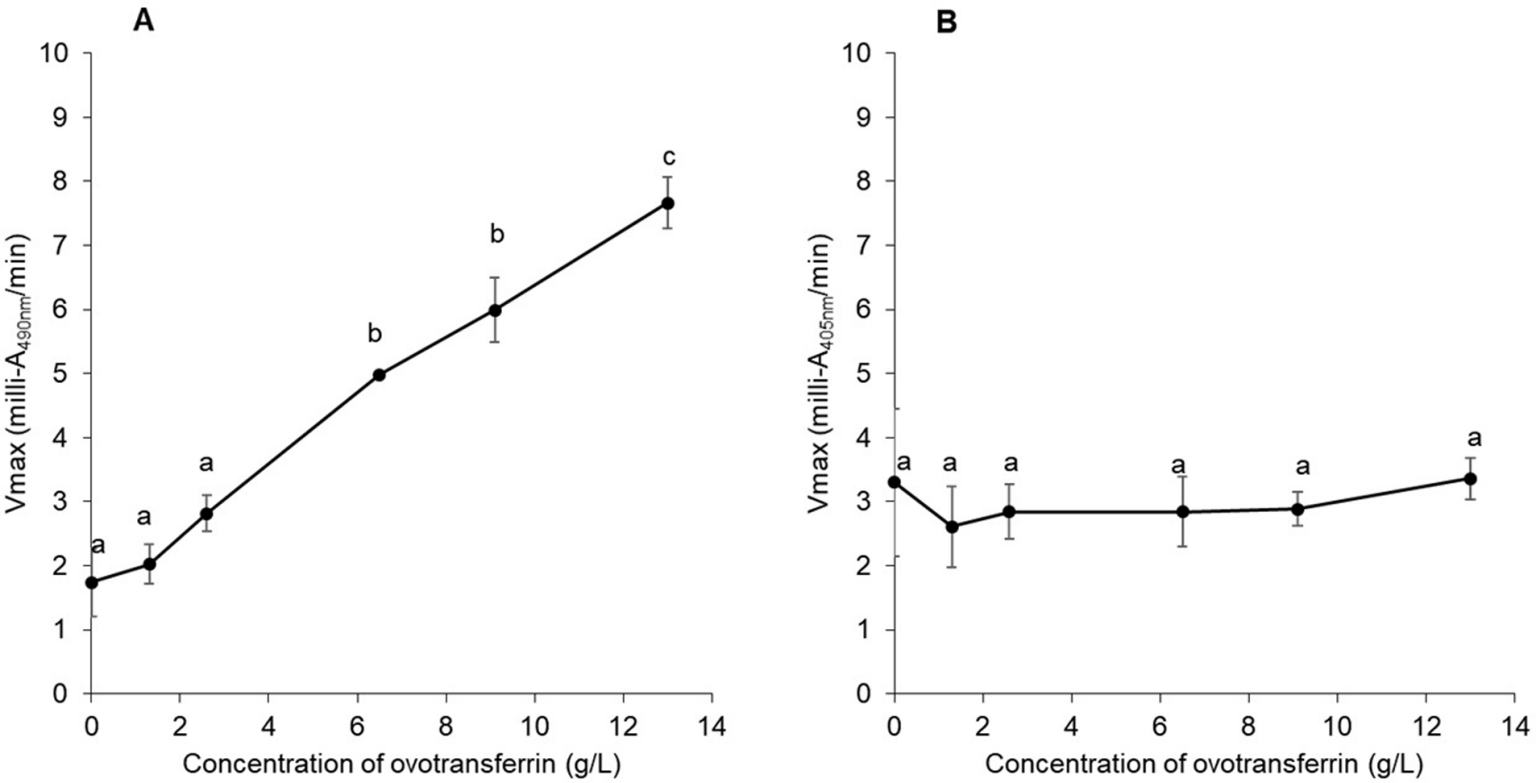
Impact of apo-OT concentration on *S.* Enteritidis membrane permeabilization. Comparison of the Vmax for outer **(A)** and inner membrane **(B)** permeabilization of *S.* Enteritidis (grown to an OD_600nm_ of 0.35) exposed to apo-OT (0 to 13 g/L) in SM. Data point represents the mean of three biological experiments with three technical replicates and error bars indicate standard deviation. Significant differences between each condition were assessed using a one-way ANOVA followed by a Tukey test. The significantly different groups are identified by distinct letters, with a *p*-value <0.05.

To confirm the impact of apo-OT on the permeabilization of the *S.* Enteritidis outer membrane and the absence of any effect of apo-OT on the inner membrane, the effect of apo-OT at 0 to 13 g/L was tested in SM (Figures 4A,B). Results show that the *β*-lactamase activity progressively increased with apo-OT concentration (Figure 4A) whilst *β*-galactosidase activity remained relatively unchanged as apo-OT concentration increased (Figure 4B). These results thus further support the ability of apo-OT to induce permeabilization of the outer membrane of *S.* Enteritidis at 30°C. The strongest outer-membrane permeabilization effect was obtained at the highest apo-OT concentration employed which corresponds to the concentration (13 g/L) found in EW. In contrast, apo-OT was not able to permeabilize the inner membrane to any notable degree.

**Figure 5:**
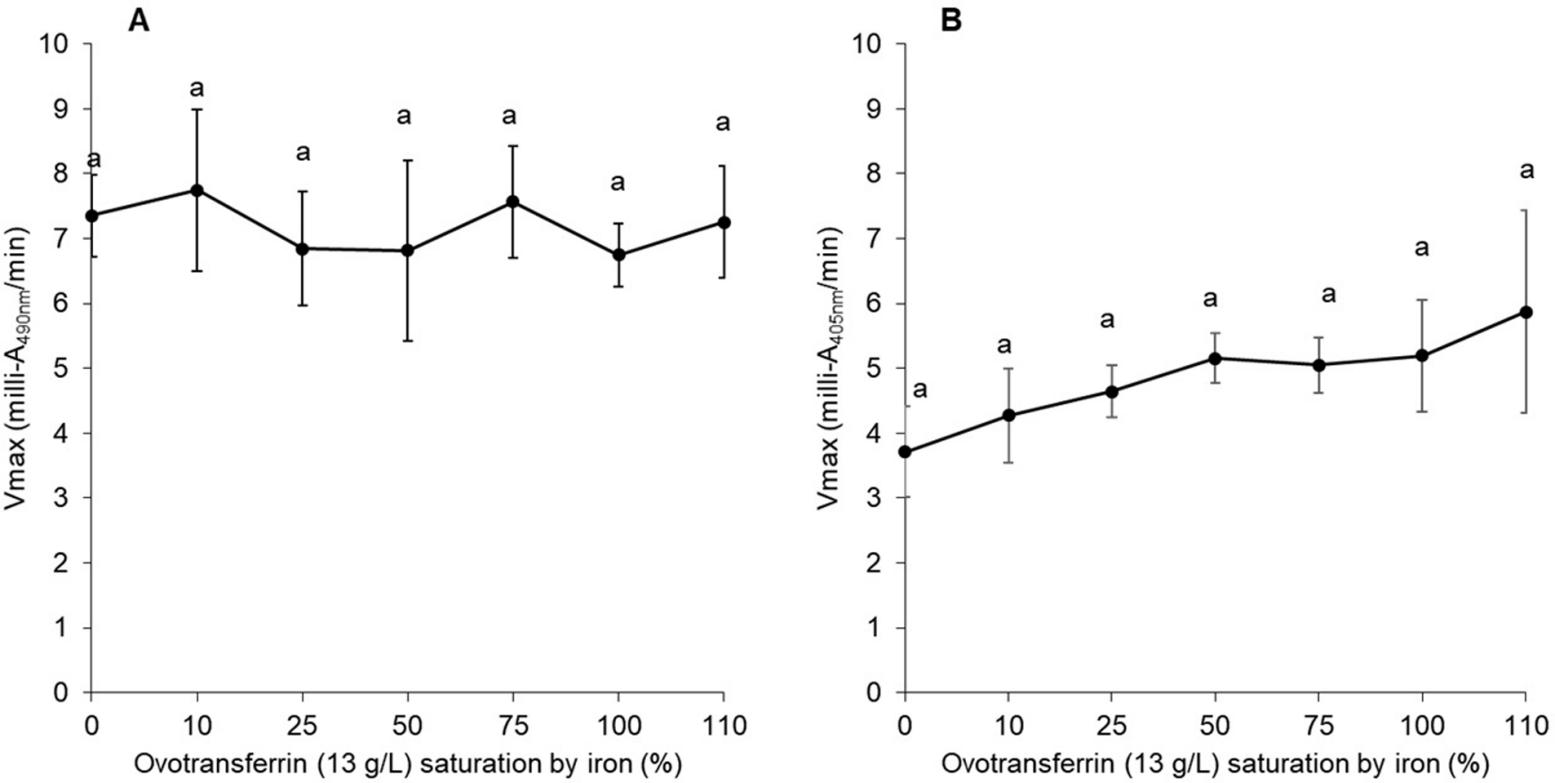
Impact of OT iron-saturation on *S.* Enteritidis membrane permeabilization. Comparison of the Vmax for outer **(A)** and inner membrane **(B)** permeabilization of *S.* Enteritidis (grown to an OD_600nm_ of 0.35) exposed to a range of OT (13 g/L in SM) saturation (from 0 to 110%) with iron citrate. Each error bar represents an average of three biological experiments with three technical replicates. Significant differences between each condition were assessed using a one-way ANOVA followed by a Tukey test. The significantly different groups are identified by distinct letters, with a *p*-value <0.05.

To explore the role of the iron-chelating activity of OT, the impact of OT saturation by iron on the permeabilization of the outer and inner membranes of *S.* Enteritidis was investigated at 30°C (Figure 5). For this purpose, OT (at 13 g/L, the concentration found in EW) was 0 to 110% iron-saturated using iron citrate, in SM. The results indicate that the ability of OT to disrupt the outer membrane is independent of its degree of iron-saturation (Vmax of 7.35 ± 0.63 and 7.25 ± 0.86 at 0 and 110% iron saturation of OT, respectively; Figure 5A). These results suggest that the metal-chelating activity and iron status of OT do not influence the ability of OT to permeabilize the outer membrane of *S.* Enteritidis. For the inner membrane, 110% iron-saturated OT induced a slightly, but not significantly, higher level of inner-membrane permeabilization than apo-OT (Figure 5B). These results suggest that apo-OT and iron-saturated OT have no notable impact on inner membrane permeabilization.

### Ovotransferrin contributes to the depolarization of *Salmonella* Enteritidis inner membrane

**Figure 6:**
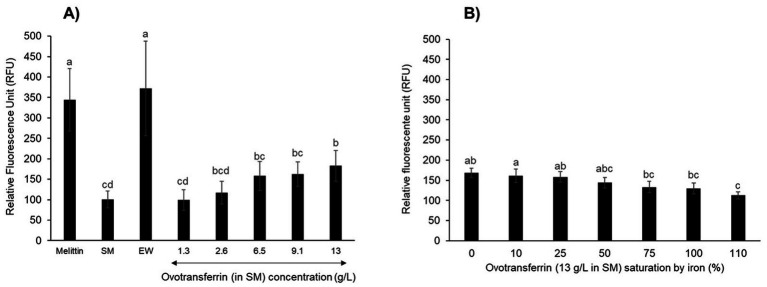
*S.* Enteritidis inner-membrane depolarization by OT. Inner-membrane depolarization of *S.* Enteritidis was monitored at 30°C after 10 min incubation with **(A)** melittin (15 μg/mL), EW, SM or apo-OT at 1.3 to 13 g/L; and **(B)** with 0 to 110% iron saturated OT (13 g/L) in SM. Each bar represents the mean of three biological experiments with three technical replicates, with standard error shown. Significant differences between each condition were assessed using a one-way ANOVA followed by a Tukey test. The significantly different groups are identified by distinct letters, with a *p*-value <0.05.

Although apo-OT was found not to induce permeabilization of the inner membrane, it remained possible that it could induce a depolarization effect. Therefore, the impact of apo-OT on the depolarization of the *S.* Enteritidis inner membrane was investigated using the DiSC_3_(5) fluorescent probe (Epand et al., 2010). Initially, depolarization of the inner membrane was measured after 10 min incubation at 30°C in response to melittin, as positive control (Figure 6A). The fluorescence reached an intensity of 344 ± 76 relative fluorescent units (RFU) and was significantly 3.6-fold greater than that obtained in response to 5 mM HEPES (pH 7.2), used as negative control (95 ± 8 RFU; data not shown). After 10 min exposure to EW, the RFU reached a similar level as with melittin (372 ± 117 RFU), indicating the ability of EW to induce inner-membrane depolarization. However, when SM was applied in the absence of any EW proteins or peptides, the level of fluorescence achieved was low (100 ± 20 RFU, similar to the 5 mM HEPES negative control at 95 ± 8 RFU; data not shown) which indicates that EW proteins/peptides are responsible for the major depolarization effect caused by EW (Figure 6A).

*Salmonella* Enteritidis was also exposed to apo-OT in SM, at concentrations ranging from 1.3 to 13 g/L (Figure 6A). No notable depolarization effect was detected for apo-OT at 1.3 g/L (99 ± 25 RFU) with respect to that obtained with SM (100 ± 20 RFU). However, a depolarization effect was observed at higher apo-OT concentrations, which an increased effect obtained as the apo-OT concentration was raised (Figure 6A). Indeed, at the concentration found in EW (i.e., 13 g/L), apo-OT caused an 83% increase in the degree of membrane depolarization (from 100 to 183 ± 37 RFU). It should be noted that the raised depolarization effect caused by apo-OT provision (with respect to SM) only became significant when apo-OT was provided at its concentration found in EW (13 g/L). This effect of 13 g/L apo-OT was significantly lower than observed for EW (372 RFU ± 117). Thus, these findings indicate that OT is at least partly responsible for the depolarization of the inner membrane observed in response to EW, but also suggest that OT is not the only EW component involved in the EW-induced depolarization of the *S.* Enteritidis inner membrane.

To confirm these results and to explore any impact of the metal-chelation activity of apo-OT on the depolarization effect, the depolarization of the inner membrane of *S.* Enteritidis was examined in response to 0 to 110% iron-saturated OT (13 g/L) (Figure 6B). The results show that as the degree of OT iron-saturation increased, there was a corresponding reduction in the level of OT-induced depolarization of the *S.* Enteritidis inner membrane (maximum reduction of 1.5-fold; Figure 6B). However, it should be noted that only OT at 110% iron-saturation (112 ± 8 RFU) gave a depolarization effect that was significantly lower than that observed for unsaturated OT (168 ± 12 RFU).

### Ovotransferrin induces an alteration in cell-surface morphology

**Figure 7:**
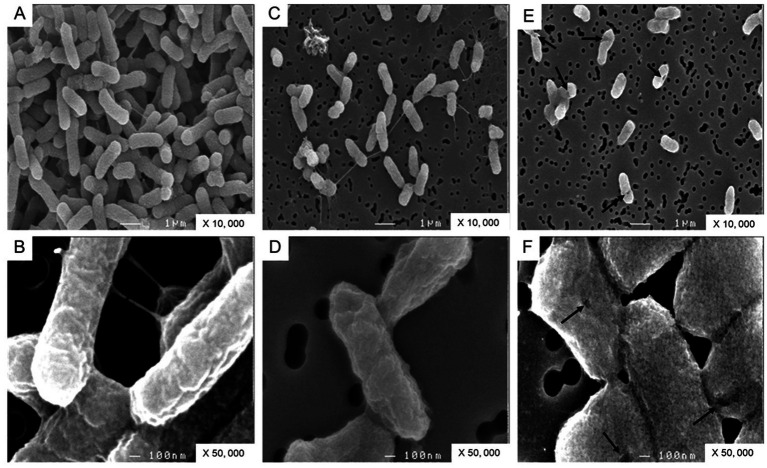
SEM analysis of *S.* Enteritidis cells. Cells were observed after 24 h incubation at 30°C (starting cell density of 7 log_10_ CFU/mL) in TSB **(A,B)**, with magnification of 10,000 and 50,000 respectively; in SM **(C,D)**, with magnification of 10,000 and 50,000 respectively; and in SM with apo-OT at 13 g/L **(E,F)**, with magnification of 10,000 and 50,000, respectively. The arrows indicate the deformations and/or pitting present on the surface of some cells.

To confirm the impact of OT on the *S.* Enteritidis envelope, the cell surface was examined by SEM. The cells were significantly shorter in SM (1.39 +/− 0.30 μm) and in SM + OT (1.24 +/− 0.35 μm) in comparison with their length in TSB (1.56 +/−0.32 μm). *S.* Enteritidis cells propagated in TSB display a relatively smooth and intact surface (Figures 7A,B). Those propagated in SM possessed a more corrugated surface although this effect was minor and the surface appeared intact (Figures 7C,D). After incubation in SM with apo-OT (13 g/L), the cells seemed more compact, displayed a granular surface and some showed shape deformation and/or pitting (Figures 7E,F). However, no evidence of cell lysis or cell debris was observed. These results thus indicate that apo-OT (at 13 g/L) induces a major morphological change, without lysis, on the *S.* Enteritidis cell surface under EW conditions.

## Discussion

Despite the antibacterial activity of EW, eggs are often sources of food poisoning which is largely caused by *S.* Enteritidis contamination (European Food Safety Authority and European Centre for Disease Prevention and Control, 2024). Among the various antimicrobial proteins of EW, OT exerts the major anti-*Salmonella* activity which is achieved through its iron-chelating activity (Baron et al., 2016). In addition to their capacity to chelate iron (and other metals), members of the transferrin family are also known to induce bacterial membrane damage (Aguilera et al., 2003; Ellison et al., 1988). Here, for the first time, the impact of OT on *S.* Enteritidis membranes under EW conditions is addressed. This study employed a novel “synthetic medium” (SM) (Table 1) designed to mimic the pH and ionic conditions of EW whilst eliminating any interference from proteins/peptides associated with EW. The results showed that the growth of *S.* Enteritidis in SM is similar to that observed in EWF (EW lacking proteins of ≥10 kDa) (Figure 1). Further, the addition of 10% EW to SM and EWF provoked similarly high degrees (~1,000 fold) of growth inhibition (Figure 1). These findings support the application of SM as a medium suitable for studying the impact of OT on *S.* Enteritidis in EW conditions. When apo-OT was added to either SM or EWF at 10% (1.3 g/L) of levels found in EW, the growth of *S.* Enteritidis was also greatly inhibited, but to a lower degree (~100 fold) than with 10% EW (Figure 1). However, a slightly (although significant) greater inhibition was observed for apo-OT in EWF than in SM; this effect might arise from the presence of <10 kDa antimicrobial peptides in EWF (Cochet et al., 2021). Although previous work has already shown that growth of *S.* Enteritidis is inhibited by EWF with 10% EW, similarly to that seen in EW (Baron et al., 1997, 2017, 2020), the current study additionally shows that apo-OT inhibits growth in SM, i.e., under conditions mimicking EW but in the complete absence of other EW proteins or peptides. Thus, EW proteins/peptides do not appear to contribute significantly to the antibacterial activity of OT toward *S*. Enterititidis under EW-conditions.

Although the principal function of transferrin is to chelate iron, it also has the ability to chelate other metals (Tan and Woodworth, 1969). To confirm the role of the iron-binding activity of OT in mediating growth inhibition in SM, the impact of iron saturation was investigated. Saturation of OT (13 g/L) with iron at 10–50% had little impact on the OT-induced growth inhibition of *S.* Enteritidis in SM. However, when OT was iron-saturated at 75–110%, this inhibition was reversed (i.e., growth was increased ~1,000 fold; Figure 2). This finding is in agreement with other reports where the iron-chelating activity of OT has been shown to be responsible for the antimicrobial activity of EW (Garibaldi, 1970; Lock and Board, 1992; Baron et al., 1997).

In this study, the main focus was the potential impact of OT on the *Salmonella* membrane perturbation under EW-conditions. Thus, the impact of OT on membrane permeabilization was explored by measurement of the activities of periplasmic *β*-lactamase and cytosolic *β*-galactosidase in *S.* Enteritidis after exposure to apo-OT in SM and to EW. EW at 30°C was found to cause permeabilization of the *S.* Enteritidis outer membrane and, to a lesser extent, the inner membrane (Figure 3). These results corroborate previous transcriptional studies that report an induction of genes involved in the maintenance of the integrity of *S.* Enteritidis membranes under EW exposure (Baron et al., 2020; Clavijo et al., 2006; Gantois et al., 2008; Huang et al., 2019, 2020; Qin et al., 2019).

Previous studies report that OT also permeabilizes the outer membrane of *E. coli* at 37°C (Aguilera et al., 2003), and that human transferrin and lactoferrin have similar effects (Ellison et al., 1988). Consistent with these findings, the results here show that apo-OT induces outer-membrane permeabilization in *S.* Enteritidis, in a concentration-dependent manner at 30°C (Figure 4A). Indeed, when apo-OT was provided at 13 g/L (its concentration in EW) in SM, permeabilization of the outer membrane was significantly greater than that obtained with EW (Vmax of 7.2 and 4.4 milli-A_490nm_/min in Figures 3A,C, respectively). This suggests, for the first time, that the activity of apo-OT against the outer membrane may be reduced by the presence of other proteins in EW.

Since the integrity of the lipopolysaccharide (LPS) layer of the outer membrane of Gram-negative bacteria is supported by divalent cations (Ca^2+^ and Mg^2+^), it was considered possible that the outer-membrane damage induced by OT may be caused by OT’s metal-chelation activity. Indeed, chelating agents, like ethylenediaminetetraacetic acid (EDTA), have been shown to disturb bacterial outer membranes by sequestration of divalent cations (Bergan et al., 2000), and OT and other transferrin-family members may act similarly as they have the capacity to bind a range of divalent cations in place of iron (Tan and Woodworth, 1969). Further, OT in EW is predicted to be only 1.07 to 5.4% saturated by iron and thus retains a considerable residual metal-chelation capacity (Legros et al., 2021). In addition, exposure of *E. coli* to lactoferrin and transferrin is reported to cause LPS release from the outer membrane and this was inhibited by the presence of iron, indicating that this effect is metal-chelation dependent (Ellison et al., 1988). In the current study, unlike previous in work, the impact of iron saturation on the outer-membrane permeabilization of *S.* Enteritidis by OT was explored under EW conditions (using SM as a protein/peptide-free EW mimic). However, no significant change in outer-membrane permeabilization was observed (at 30°C) for *S.* Enteritidis in response to the iron-saturation of OT (Figure 5A) which indicates that the metal-chelating activity OT is not involved in outer-membrane permeabilization of *S.* Enteritidis by OT. Clearly, these results differ from those obtained for *E. coli* (Ellison et al., 1988). This discrepancy may reflect differences between the envelopes of the two bacterial species, as well as between the OT used herein, and the transferrin and lactoferrin proteins tested by Ellison et al. (1988) (e.g., pI and glycosylation status), and also between the pH conditions employed. Indeed, Ellison et al. (1988) reported that an increase of pH (from 5.5 to pH 8) caused LPS release for *E. coli*; this indicates that alkalinity can induce outer membrane instability. Thus, it is likely that the high pH (9.3) applied here resulted in reduced outer membrane stability for *S.* Enteritidis that may explain the differences in findings between the studies. In summary, the results obtained here indicate that the metal-chelation activity of OT does not play a role in the permeabilization of the outer membrane of *S.* Enteritidis by OT when investigated under EW-conditions.

In contrast to the impact of OT on the outer membrane, OT failed to mediate permeabilization of the inner membrane of *S.* Enteritidis (Figures 3B,D) at any of the concentrations tested (Figure 4B). It is therefore likely that the observed permeabilization of the inner membrane by EW (Figure 3) is contributed by EW proteins other than OT. Indeed, the ability of hen lysozyme to permeabilize both the outer and inner membrane of *E. coli* at 37°C and pH 7.4 (Derde et al., 2013) suggests a role for lysozyme in the inner-membrane permeabilization induced by EW. However, this effect has not yet been tested for *S.* Enteritidis under EW conditions and further investigations are needed to explore the impact of other EW proteins on the inner membrane. The iron supplementation of OT did not cause a notable effect on the *S.* Enteritidis inner-membrane.

As found here for *S.* Enteritidis, *E. coli* also displays permeabilization of the outer-membrane, but not the inner-membrane, upon OT exposure (Aguilera et al., 2003). Despite this lack of inner-membrane permeabilization, OT did cause a reduction of the inner-membrane potential (ΔΨ) for *E. coli* which was combined with a release of intracellular K^+^. This finding suggests that OT can gain access to the inner membrane of *E. coli* and negatively impact its integrity (Aguilera et al., 2003). Likewise, the current study revealed a strong depolarization of the *S.* Enteritidis inner membrane after exposure to EW (Figure 6A). This finding is consistent with previous work showing that *S.* Enteritidis exposed to 10% EW in EWF at 45°C also suffers inner-membrane depolarization (Baron et al., 2020), although here exposure was at 30 rather than 45°C (to avoid the bactericidal effect induced at 45°C). The previous finding that 10% EW (in EWF) induces the *psp* genes of *S.* Enteritidis at 45°C (Baron et al., 2020) is consistent with the depolarization of the *S.* Enteritidis inner membrane by EW. Like EW, OT induced a clear depolarization of the inner membrane of *S.* Enteritidis and this was directly dependent on the OT concentration (Figure 6A). However, the OT-depolarization effect only reached significance at the maximum concentration explored (13 g/L; Figure 6A). Similarly, the OT-induced dissipation of the membrane potential of *E. coli* was also found to be dependent on OT concentration (Aguilera et al., 2003). Depolarization of the cytoplasmic membrane by OT (at 13 g/L) has also been reported for the Gram-positive bacterium, *B. cereus* (Baron et al., 2014). However, it should be emphasized that the findings reported herein are the first time that an impact of OT on the *Salmonella* membrane potential has been identified under EW-conditions in the absence of potential inference from other EW proteins/peptides.

The electrical potential across the inner membrane can be dissipated by a perturbation of the membrane that allows the flow of charge without necessarily permitting the passage of small molecules, such as ONPG (used to measure inner-membrane permeabilization; Epand et al., 2010). This may well explain how OT induces inner-membrane depolarization, as observed here, even in the absence of any apparent inner-membrane permeabilization by OT (Figures 3B,D). OT is predicted to be negatively charged at pH 9.3 (Legros et al., 2021), so it be can hypothesizes that, at alkaline pH, more OT may accumulate in the periplasm, gaining greater access to the inner membrane, resulting in an enhanced effect on the membrane potential. This hypothesis is supported by a lower inner-membrane depolarization when OT is saturated with iron (Figure 6B). The saturation of OT by ferric iron is reported to lower the overall negative charge of OT (Yoshioka et al., 1966) which might influence its ability to disrupt the potential of the inner membrane. EW induced a two-fold stronger increase in depolarization than OT (Figure 6A). This indicates that other EW proteins or peptides also contribute to the EW-induced membrane depolarization effect observed for *S.* Enteritidis. EW proteins such as lysozyme, defensins and bactericidal permeability-increasing proteins (BPI) (Baron et al., 2016), or unknown peptides, may thus also contribute to the observed depolarization effect of EW.

SEM showed that apo-OT, at the concentrations found in EW (13 g/L), induces a morphological change in the *S.* Enteritidis cell surface, which corroborates the permeabilization and depolarization results. It is important to note that despite the membrane perturbations observed, no cell lysis or cellular debris was observed. This is in agreement with our growth results: *S.* Enteritidis is able to survive in both EW and SM with OT at 30°C (i.e., cell number increased by 0.3 and 0.4 log_10_ CFU/mL, respectively). Thus, OT does not provoke bulk bacterial lysis and so, at 30°C, *S.* Enteritidis is able to cope with the membrane stress induced by EW and/or OT. Indeed, several studies have highlighted the induction of genes involved in the maintenance of *S.* Enteritidis membrane integrity in response to EW exposure (Baron et al., 2020; Clavijo et al., 2006; Gantois et al., 2008; Huang et al., 2019, 2020; Qin et al., 2019).

**Figure 8:**
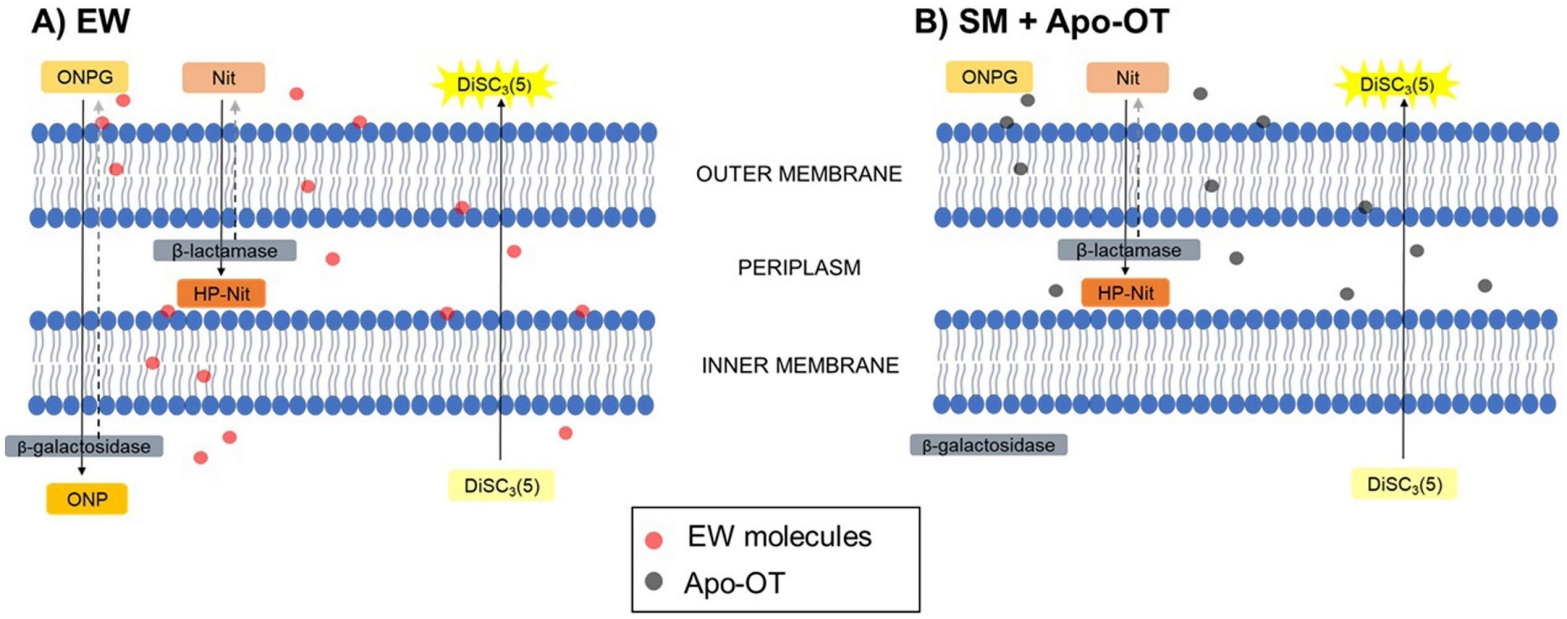
Hypothetical action of EW and OT on the membranes of *S.* Enteritidis. Abbreviations are as follow: Nit (nitrocefin) and HP-Nit (HP-nitrocefin) are the substrate and product of *β*-lactamase, respectively; ONPG and ONP are the substrate and product of *β*-galactosidase, respectively; DiSC_3_(5) is the potentiometric probe. After exposure to **(A)** EW, Nit was internalized into the periplasm and hydrolyzed into HP-Nit by the *β*-lactamase, indicating a permeabilization of the outer membrane of *S.* Enteritidis. In the same manner, ONPG was internalized into the cytoplasm and hydrolyzed into ONP by the *β*-galactosidase, indicating a slight permeabilization of the inner membrane. Furthermore, the depolarization of the inner membrane was also indicated by the extracellular release of the fluorescence probe DiSC_3_(5). After exposure to **(B)** SM with OT, Nit was internalized into the periplasm and hydrolyzed into HP-Nit, indicating a permeabilization of the outer membrane, however, ONPG was not internalized into the cytoplasm. Independently of the permeabilization of the inner membrane, DiSC_3_(5) was released, showing the ability of OT to induce inner-membrane depolarization.

In summary, the results reported here indicate a role for OT in inducing *S.* Enteritidis membrane damage, under conditions mimicking those of EW, at 30°C. The hypothetical action of EW and OT on *S.* Enteritidis membranes is depicted schematically in Figure 8. EW permeabilizes the *S.* Enteritidis outer membrane and OT appears responsible for this activity. This may allow other EW molecules to subsequently access the periplasm and induce depolarization of the inner membrane, and, to a lesser extent, permeabilization of the inner membrane. OT is not able to permeabilize the inner membrane but, possibly due to its overall negative charge at pH 9.3, OT contributes to the depolarization of the inner membrane. These findings thus provide new insight into the role and mechanism of OT in countering *S.* Enteritidis in EW. Such understanding of this key EW protein is relevant to the egg-production sector and to food safety due to the contribution of eggs and egg products to foodborne outbreaks.

EW has a specific ionic composition and an alkaline pH (that increases during egg aging/storage) that can be expected to influence the antimicrobial properties of EW proteins. This investigation introduces a defined EW model medium mimicking these particular conditions which therefore allows studies of the impact of OT on *S.* Enteritidis under controlled EW conditions. In future work, this synthetic medium could be employed to investigate the impact of other EW proteins or variation in ionic composition on *S.* Enteritidis. Such investigations may indicate modifications to the hen diet (that could result in changes to the ionic composition of EW; Schiavone and Barroeta, 2011) and/or egg-storage practices (which can impact EW pH and ion flux between yolk and EW through vitelline membrane degradation) (Réhault-Godbert et al., 2019) that disfavor *S.* Enteritidis contamination of eggs and thus enhance egg safety.

## Data Availability

The original contributions presented in the study are included in the article/supplementary material, further inquiries can be directed to the corresponding author.
